# Excipient-excipient interactions in the development of nanocarriers: an innovative statistical approach for formulation decisions

**DOI:** 10.1038/s41598-019-47270-w

**Published:** 2019-07-24

**Authors:** Viviane Lucia Beraldo-de-Araújo, Anderson Beraldo-de-Araújo, Juliana Souza Ribeiro Costa, Ana Carolina Martins Pelegrine, Lígia Nunes Moraes Ribeiro, Eneida de Paula, Laura Oliveira-Nascimento

**Affiliations:** 1 Department of Biochemistry and Tissue Biology, Biology Institute, State University of Campinas, Brazil, Rua Monteiro Lobato, 255, Campinas, SP 13083-862 Brazil; 2 Pharmaceutical Technology Laboratory, Faculty of Pharmaceutical Sciences, State University of Campinas, Brazil, Rua Candido Portinari, 200, Campinas, SP 13083-871 Brazil; 3 Center for Natural and Human Sciences, Federal University of ABC, Santo André, 05508-090 Brazil

**Keywords:** Drug delivery, Nanoparticles

## Abstract

Excipient interaction has become essential knowledge for rational formulation design of nanoparticles. Nanostructured lipid carriers (NLCs) include at least three types of excipient, which enhance excipient interaction possibilities and relevance. The present article introduces an alternative approach for evaluating a great number of excipients with few samples, using NLC as a model delivery system. This approach is based on two sequential experiments using Hall-2 experimental design and analysis of excipient interactions in respect to their physicochemical properties by multilevel statistics. NLCs were prepared using a hot emulsification-ultrasonication method with lidocaine and nine excipients (solid lipids, oils and surfactants). The evaluated parameters were z-average size (DLS), dispersity (DLS), zeta potential (electrophoretic mobility) and entrapment efficiency (HPLC). Cetyl palmitate, beeswax, castor oil, capric/caprylic acid and polysorbate 80 all presented larger effects amongst the studied factors as well as a clear pattern of synergistic interactions. Following the verified trends, we produced an optimized NLC that exhibited all desirable physicochemical characteristics and a modified drug release profile. Our results demonstrate the methodology’s robustness, which can be applied to other nanoparticles and establish a cost-effective excipient evaluation.

## Introduction

Nanostructured lipid carriers (NLCs) are the second generation of solid lipid nanoparticles (SLN), widely used as biodegradable and safe delivery systems for hydrophobic drugs and bioactive substances^1–3^. They are submicron particles of a mixed solid-liquid lipid core coated with surfactants; the carried substance generally being located within the lipid core. In order to obtain reproducible and stable NLCs, they should present low particle size dispersity, high zeta potential in module and constant size range^4^. To manipulate these properties, one could alter critical process parameters or formulation parameters. Critical formulation parameters of NLCs include lipid type, amount and melting point; core crystallinity; drug and surfactant properties^5^. Therefore, to perform a rational formulation design, it is crucial to know which excipients interact with each other and change physicochemical attributes.

Some researchers assessed both qualitative and quantitative influences of the excipient type/quantity for NLCs. Most of them performed small and isolated experimental full designs with few excipients, which aids formulation improvement but not interaction screening^6–10^. When screening is prioritized, there is a plethora of design available^11^, but the Plackett-Burman design is probably the most common^12^. This design works for robustness assessments that check whether slight factor modifications influence outputs. Yet, when factors interact with each other, individual outcomes are indistinguishable from interactions^12^. Another important type of approach is the application of mixture designs to formulation development, especially for pharmaceutical formulations^13^. Nonetheless, in the context of many factors, mixture designs tend to be hard to analyse. Finally, in the case of fractional factorial design, another design type used regularly, main individual factors can be distinguished from others, but two-factor interactions are generally confounded^14^. Due to the complex nature of NLCs, it is likely that excipients will interact with each other, making it impossible to separate this from the excipient’s individual behaviour using the techniques described.

In this article, we intent to explore Hall’s design to overcome the mentioned limitations. As a non-regular design with two levels, it permits the combination of up to 15 factors in 16 runs, without full aliasing of two main factors^15^. However, this has a cost: Hall’s design is a relatively high-risk design because it has strong aliasing of two factor interactions with multiple additional two factor interactions (see Hall´s DoE correlation matrix on Supplementary Fig. S1). Given that, we present a method based on Hall´s design to analyse excipient interaction on physicochemical outcomes that permits a robust analysis and allows us to systematically deal with the risk associated to Hall’s design. We study excipient interaction on physicochemical outcomes of NLCs loaded with lidocaine, but, since two-factor interactions are likely to appear in any significant formulation, this approach applies to any other excipient screenings, from the pharmaceutical to the cosmetics and food fields.

The method begins with pre-formulation studies to evaluate the viability of each excipient concerning lidocaine solubility/compatibility and excipient purity. Five natural liquid lipids (castor, sesame, cottonseed, corn, and capric/caprylic oils), two natural solid lipids (beeswax and cetyl palmitate) and two surfactants (poloxamer 188 and polysorbate 80) were evaluated. The individual assessments were followed by an NLC fabrication to check physicochemical outputs. In the second stage, we implemented two sequential Hall-2 design experiments with 10 factors. In our method, the excipient levels change throughout the experiments to assess the main effects and two-factor interactions. This helps to discover tendencies that can serve as criteria for choosing the excipients and for developing optimized formulations. After performing the experiments, we analysed some physicochemical outputs of the resultant NLCs (z-average size, dispersity, zeta potential and entrapment efficiency). Then, multilevel analysis was applied to determine variance throughout the two experiments, followed by the formulation of linear models of effects and interactions. To build graphs of excipient interaction, we applied Wu-Hamada’s formula of conditional effects^16^. Finally, we fabricated an optimized NLC, according to the output evaluation, to confirm the statistical assessments.

## Results

### Lidocaine solubility and partition coefficient

First, we need to guarantee complete drug solubilization in all possible formulations. Table 1 shows that all lipids solubilized more than 100 mg/mL of LD (liquids) or more than 0.2 mg/mL of LD (solids); both mass proportions are the low mass levels proposed in our DoE. Surfactants augmented LD solubility in water at least two-fold. According to the United States Pharmacopeia (USP) solubility definition, LD was very soluble in solid lipids, freely soluble in oils and slightly soluble in water and surfactants^17^. LD solubility presented the following order: liquid lipids: CA=CC > SO=CO > CS; surfactants: PS > KO; solid lipids: BW > CP.

**Table 1:** Solubility of lidocaine (LD) in the chosen excipients.

Excipient	Solubility	Partition coefficient (mean ± SD)
*Solid lipids (mg/mg)*		
BW	7	1.11 ± ND
CP	4	0.80 ± 0.02
*Oils (mg/mL)*		
CA	260–280	1.61 ± 0.01
CC	260–280	1.29 ± 0.02
SO	160–180	1.13 ± 0.03
CO	160–180	1.18 ± 0.03
CS	120–140	1.17 ± 0.03
*Surfactants and water (mg/mL)*		
PS 1%	5	—
KO 1%	4	—
Deionized water	2	—

(BW = beeswax, CP = cetyl palmitate, CA = castor oil, CC = capric/caprylic oil, SO = sesame oil, CO = corn oil, CS = cottonseed oil, PS 1% = polysorbate 80 1% (V/V) in water, KO 1% = poloxamer 188 1% (w/V) in water). The solubility range of LD in oils is due to determination method and signifies the amount that is solubilized (lower number) and the amount that is not completely soluble in each oil (higher number). The partition coefficient of LD in solid and liquid lipids are expressed as mean ± standard deviation). The experiment was carried out in duplicate.

Partition coefficients were also determined because of their influence on encapsulation rates. Lipids with higher or lower LD solubility (Table 1) also presented higher and lower partition coefficients, respectively. Partition coefficient values of LD presented the following order: CA > CC > SO=CO = CS=BW > CP.

### Excipient impurities and NLC characterization

**Figure 1:**
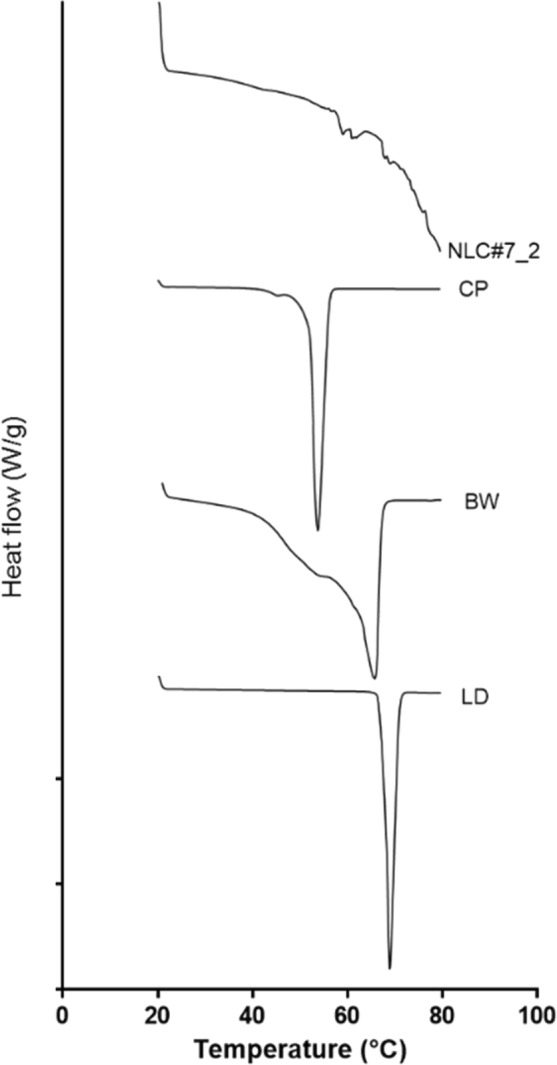
Thermal analysis (DSC) of solid lipids (Cetyl Palmitate, CP and Beeswax, BW), Lidocaine (LD) and NLC#7_2 (from experiment 2). Melting peaks: LD = 68.87 °C, CP = 53.82 °C, BW = 65.7 °C, NLC#7_2 = two major peaks, 58.89 and 67.77 °C. Analyses were carried out at a heating rate of 5 °C per minute and N_2_ atmosphere.

Impurities may decrease LD stability and mask excipient interaction analysis. Therefore, thermal analyses were performed to confirm the purity of super-refined excipients, besides lidocaine encapsulation. Figure 1 shows that CP presented one melting peak of a β polymorph state, the most stable crystalline form of lipids^2^. LD also presented one narrow melting peak, which indicates purity and meets the range determined by USP for drug identification^18^. BW presented a large peak due to its complex mixture of organic compounds, which is in accordance with the reported range^9^. Thermal analyses of liquid lipids presented no peaks in the 20–80 °C range, which is expected for liquid samples at ambient temperature. We then fabricated one NLC composed of all excipients, based on excipient quantities from univariate tests (data not shown) to outline experiments 1 and 2 and perform output analyses. The NLC (number 7 from exp 2, Supplementary Table S2) presented peaks at two different regions: one at 58.89 °C, probably from BW, and another at 67.77 °C, corresponding to non-encapsulated LD. The small free LD peak found in NLC analysis corroborates with good entrapment efficiency (74.1%). The evaluated formulation also presented good physicochemical characteristics (z-average 219 ± 1 nm; PDI 0.24 ± 0.01; zeta potential −41 ± 0.6 mV and drug loading 2.2%). TEM micrographs revealed intact NLCs (Supplementary Fig. S3), with spherical shapes and defined borders. Also, the sizes of the nanoparticles were consistent with those determined by DLS (data not shown).

### Data of NLCs physicochemical properties

The statistical analyses of the data from experiments 1 and 2 were performed with z-average size (ZA), dispersity (PDI), zeta potential (ZP) and entrapment efficiency (EE) responses (the mean samples on Supplementary Tables S3 and S4). Table 2 displays the means, standard deviations and medians of those responses. The ANOVA used to assess intercept variance between experiments (GLS *versus* LMM) showed no significant influence on response behaviour with respect to the means of all samples. The Coefficient of determination (R^2^) and F statistics from GM and GM applied to exp 2 were also presented. Consequently, we can analyse the variation across the experiments with a general linear regression model. Since all the formulations reached desirable ZP values (smaller than -34.9 mV), it is unnecessary to restrain the factor levels that contributed to influence ZP; therefore, no GM was generated for this output.

**Table 2:** Comparison of physicochemical parameters in experiments 1 and 2.

Parameter	Exp 1	Exp 2	ANOVA (GLS *vs* LMM)	GM	GM applied to Exp 2
Z-average (nm)	330 ± 43^a^	315 ± 52^a^	*0.99* ^c^	*R* ^2^ * = 0.80*	*R* ^2^ * = 0.95*
	335^b^	318^b^		*F (8*,2*3) = 11.54*	*F (8,7) = 15.83*
PDI	0.26 ± 0.06^a^	0.22 ± 0.06^a^	*0.4*2^c^	*R* ^*2*^ * = 0.84*	*R* ^*2*^ * = 0.94*
	0.25^b^	0.21^b^		*F (9,22) = 12.45*	*F (9,6)* = *10.47*
Zeta potential (mV)	−45.9 ± 4.0^a^	−46.6 ± 4.8^a^	0.99^c^	—	—
	−47.5^b^	−48.0^b^			
Entrapment Efficiency (%)	70 ± 6^a^	74 ± 4^a^	*0.62* ^c^	*R* ^*2*^ * = 0.85*	*R* ^*2*^ * = 0.98*
	72^b^	74^b^		*F (14,17) = 6.84*	*F (10,5)* = *27.94*

^*a^aritmethic mean ± standard deviation; ^b^median; ^c^p-value.

### Main effects and interactions in Z-average

**Figure 2:**
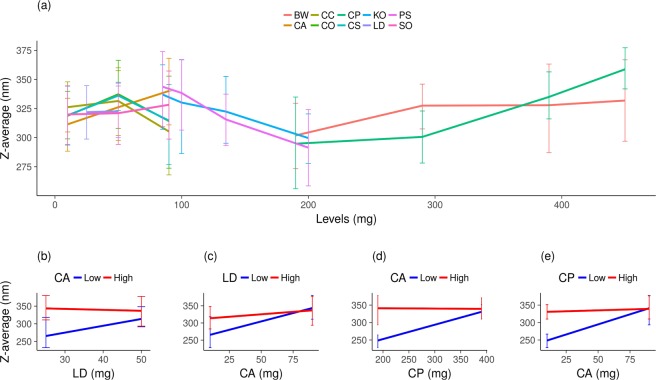
ZA distribution from experiment 1 and 2 (Hall experimental design, Supplementary Tables S1 and S2) at low and high levels of each excipient. Error bars correspond to the standard deviation of the samples’ mean of each experiment. The level values of each experiment are in (**a**); interaction of LD on CA in z-average of exp 2 (**b**); interaction of CA on LD in z-average of exp 2 (**c**); interaction of CP on CA in z-average of exp 2 (**d**); interaction of CA on CP in z-average of exp 2 (**e**).

Figure 2(a) displays the behaviour of ZA throughout the two experiments. CA (p = 0.00004), CP (p = 0.000003), PS (p = 0.02) and SO (p = 0.0005) acted to increase ZA (GM, Supplementary information p. 10). The interactions observed are described in GM applied to exp 2 (Supplementary information p.10), of which those with the greatest effects were discussed further.

Among the liquid lipids, CA had significant interaction with CP (p < 0.003) and LD (p = 0.003). As displayed in Fig. 2(b,c), CA presented a significant interaction with LD: when CA was at the highest level, it made ZA greater (Fig. 2(b)). Irrespective of the level of LD, CA acted to increase ZA or maintain it higher (Fig. 2(c)). The distinguished behaviour observed for CA was also an effect of its interaction with CP: CA at high level and CP at low level interact to increase size just like the high-level CP profile (Fig. 2(d,e)).

The interaction between PS and KO was significant enough to decrease ZA (p = 0.002). Their action is collinear with PS prominence (*p* = *0.0*2), which interacted with CS and CO as well (section 2.6) - this explains why PS possesses a positive coefficient in the model despite its clear action to decrease ZA (Supplementary information p.10).

### Main effects and interactions in dispersity

**Figure 3:**
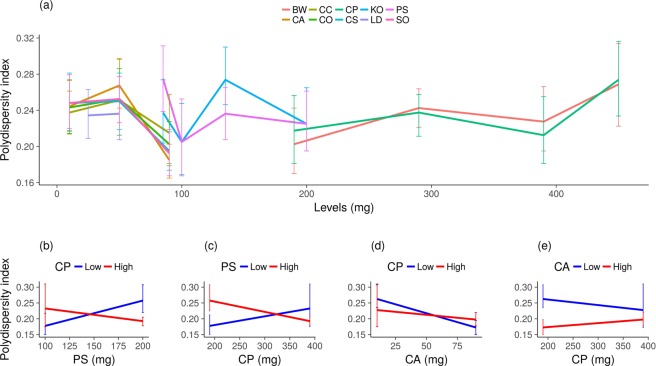
Polydispersity index (PDI) distribution from exp 1 and 2 at low and high levels of each excipient (**a**). Error bars correspond to the standard deviation of the samples’ mean value of each experiment. The level values of each experiment are in Supplementary Tables S1 and S2; interaction of PS and CP in PDI of exp 2 (**b**); interaction of CP and PS in PDI of exp 2 (**c**); interaction of CA and CP in PDI of exp 2 (**d**); interaction of CP and CA in PDI of exp 2 (**e**).

Figure 3(a) exhibits the PDI behaviour throughout both experiments. The factors that influenced ZA were almost the same as those that influenced PDI. The main effects were from PS (to increase PDI, p = 0.0000007), CA and BW (both to decrease PDI, p = 0.0003 and p = 0.000005, respectively). Concerning interactions, the greatest effects were found for interactions of PS and CP (*p* = *0.0005*) and CP and CA (p = 0.01). If CP was at a high level, PS acted to decrease PDI, but when CP was low, PS increased PDI (Fig. 3(b)). Contrarily, if PS was at a high level, CP decreased the PDI, but if PS was low, CP increased PDI (Fig. 3(c)). CP also interacted with CA: if CP was at a high level, CA had a small effect; but when CP was low, CA decreased PDI (Fig. 3(d)). However, if CA was at high level, CP had a small effect in increasing PDI, but CP decreased it if CA was low (Fig. 3(e)). Therefore, CP, CA and PS presented interactions.

Despite BW acting to decrease PDI, its interaction with two excipients, CP and KO, were both significant enough to increase it (p = 0.002 and p = 0.03, respectively). As their effects were smaller than the others (supplementary information p.12), we focused on the following interactions.

### Main effects and interactions in Entrapment efficiency

**Figure 4:**
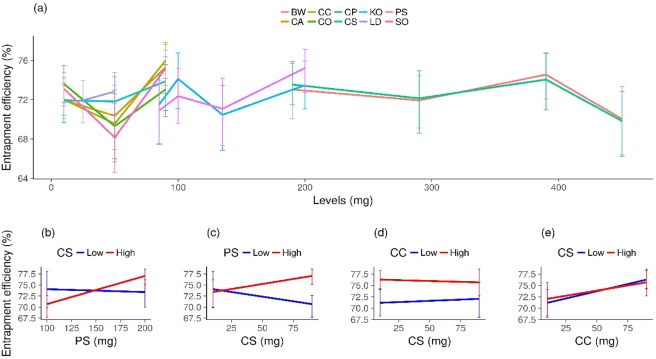
Entrapment efficiency (EE) distribution from exp 1 and 2 at low and high levels of each excipient (BW = beeswax, CA = castor oil, CC = capric/caprylic oil, CO = corn oil, CP = cetyl palmitate, CS = cottonseed oil, KO = poloxamer 188, LD = lidocaine, PS = polysorbate 80, SO = sesame oil). Error bars correspond to the standard deviation of the samples’ mean value for each experiment. The level values of each experiment can be found in Supplementary Tables S1 and S2 (**a**); interaction of PS and CS in EE of exp 2 (**b**); interaction of CS and PS in EE of exp 2 (**c**); interaction of CS and CC in EE of exp 2 (**d**); interaction of CC and CS in EE of exp 2 (**e**).

Figure 4(a) shows that entrapment efficiency (EE) did not demonstrate a clear pattern of excipient behaviour. GM indicated that CA was the only factor that increased EE (*p* = *0.01*). On the other hand, GM indicated a negative coefficient for PS (Supplementary information p.14), contrary to the tendency seen in Fig. 4(a), which may be associated with the excipient’s interactions. PS interacted with CS (*p* = *0.007*) and CP (*p* = *0.001*). We presented (Fig. 4(b–e)) the two major interactions, namely, PS and CS (*p* < *0.05*) as well as CC and CS (*p* < *0.0*2). Figure 4(b) indicates that PS has an effect of increasing EE when CS is at its highest level. However, CS increases EE only when PS is at a high level, and slightly decreases it when PS is at a low level. In Fig. 4(d) CS has no effect on CC levels, and EE is directly proportional to the CC level. This is confirmed in Fig. 4(e), where CC increased EE irrespective of the CS level.

### Optimized NLC with BW, CA and PS

**Figure 5:**
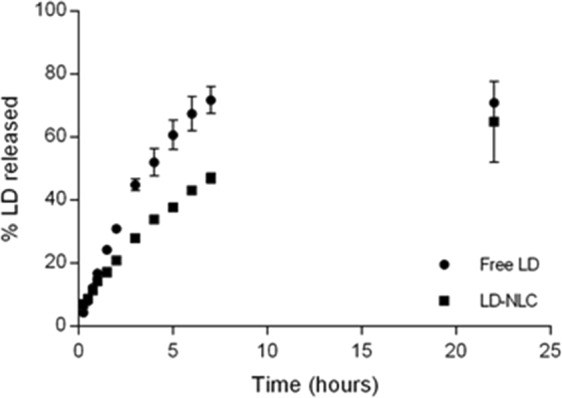
*In vitro* drug release profile of free LD and NLC-loaded LD.

In order to confirm the excipient effects, a new NLC was made containing BW, CA, PS and LD (700 mg, 300 mg, 300 mg and 50 mg, respectively). The choice of these excipients relied on the fact that they strongly contributed to decrease PDI and increase EE, both of which are desirable in most applications. The physicochemical properties of this optimized NLC were: ZA (267 ± 7) nm, PDI (0,17 ± 0,02), ZP (-41 ± 2) mV and EE (80.1 ± 0.1) %, which successfully confirmed the statistical analysis. In addition, we performed an experiment to determine the *in vitro* drug release profile of the free LD and NLC-loaded LD. Figure 5 illustrates the results obtained for a 22-hour period. Initially, in the first hour, we can almost see an overlap between the replicates from LD-NLC and the free drug, which may be due to the diffusion of the non-encapsulated LD portion across the membrane barrier. From the second hour on, the releases are quite different, proving that NLC modified the LD release profile. Nearly 50% of LD diffuse through the membrane after 4 hours (free LD), but the same amount took 7 hours for LD-NLC. At 22 hours, the differences were not distinguishable (approx. 70% release).

## Discussion

Pre-formulation studies were essential to confirm high purity levels and compatibility of excipients with lidocaine. In general, ZA size was influenced by the amounts of all excipients, except for liquid lipids in isolation. In accordance with Teeranachaideekul^10^, liquid lipids did not affect particle size in the studied ranges in isolation. Solid lipids contribute to increase lipid phase viscosity at high wax concentrations^19^, including CP^4^, which affects mixing during NLC production (via the hot homogenisation method) and leads to particle aggregation and size increase, also increasing size dispersity^4,9^. Liquid lipids are not inert, and may have interactions with other excipients, changing nanoparticle size. For example, this happened with CA when it interacted with LD and CP and contributed to increase ZA size on all levels, except when CA and CP were at low levels. This CA action may be explained by Hu *et al*.^20^, who found smaller nanoparticles from the use of less viscous oils (CA was the most viscous oil that we used). CA also highlighted its influence amongst the liquid lipids, which decreased PDI at high levels. Besides that, a diminishment of both ZA and PDI by increasing liquid lipids was observed, which stimulated the formation of nanoparticles with a narrow size distribution due to size reduction^5,21^.

Surfactants acted to decrease ZA, which is in accordance with Helgason *et al*.^22^ statement that the increase in SLN particle size is accentuated with low surfactant concentrations (they worked with 10% of solid lipid and a variation between 1–5% of tween 20 (w/w)). According to Helgason *et al*., this occurs because the particle surface is less covered by the surfactant and, therefore, there is an increased probability of particle-particle interactions happening^22^. The relationship between solid lipids and surfactants depends on the level and type of each factor. Figure 3(b,c) prove this point. This shows that Gonzalez-Mira *et al*.^23^ assertion that surfactants alone can contribute to lower PDI values is not a general rule. They concluded this (studying KO) because their inputs were proportions of total lipids and surfactants, which do not provide information about interactions among excipients.

Our EE results with regard to liquid lipids agreed with Pathak *et al*. assertion that the entrapment increase is dependent not only on the solubility of the drug in the carrier, but also on the amount of liquid lipid^5^. However, special care relating to a high increase in the liquid lipids amount must be taken, because it may influence the immobilization capacity of the solid lipid and decrease EE^24^.

The ZA size analysis indicates that, to choose among the levels of each factor, it is necessary to consider the formulation applicability and its desirable size. For example, if one is developing an NLC for a parenteral application, NLC should be between 100–200 nm^25^. In this case, according to the ZA analysis, it would be better to choose CP and CA at low levels and surfactants at high levels. In the case of PDI, lower values are always desirable, so CP in low levels and CA in high levels would be appropriate. Despite the absence of a general pattern on EE, the analysis permitted us to conclude that we should use PS at higher levels. Moreover, because PS interacted with CP to increase ZA, if we want small particles with high EE, it is better to use BW rather than CP, with PS.

Finally, optimized NLC presented desirable physicochemical outputs, compatible with the experimental design used for excipient screening. *In vitro* release kinetics showed a sustained drug release profile, confirming that the final formulation not only solubilized the drug, but also prolonged its release. We did not intend to cover the influence of each excipient in the LD release kinetics. Noteworthy, Leng *et al*. incorporated LD into SLN and described different drug release kinetics depending on excipients: the equivalent to 50% of LD released in our experiment was found by them after approximately 2 h from SLN with stearic acid, 3 h from SLN with monostearin, and 6 h from SLN with glyceryl palmitostearate^26^. Given this, our LD-NLC release was similar to LD-SLN with glyceryl palmitostearate.

## Conclusion

As shown by our results, excipient interactions do change physicochemical outputs and are essential to performing a rational NLC formulation. The statistical models were appropriate and elucidated the behaviour of excipient interactions. Our analysis exhibited that CA, CP, CC and PS were the highlighted excipients along the different responses, and we outlined some general patterns in order to make optimal NLCs. The interactions among PS, CA and CP were decisive for ZA. PS, CA and CP were also the main factors that influenced PDI. Regarding EE, the interaction among liquid lipids was crucial, with special emphasis on CS and CC. In addition to the statistical model values, we achieved an optimal formulation of NLC-loaded LD fabricated with highly purified lipids, an ideal feature for future *in vivo* parenteral studies. Our method can also be applied to other drug delivery systems as well, with the advantage of a reduced number of samples and, therefore, a cost-effective method for pharmaceutical formulations.

## Methods

### Materials

Super Refined™ excipients were generously donated by Croda do Brasil: castor oil (CA), sesame oil (SO), cottonseed oil (CS), corn oil (CO), polysorbate-80 (PS), beeswax (BW), Crodamol™CP-PA (CP). Liponate^®^ GC (Capric/caprylic oil, CC) was gently donated by Lipo do Brasil. Poloxamer 188 (Kolliphor^®^ 188, KO), acetonitrile and lidocaine (LD) base were purchased from Sigma.

### Selection of lipids

The previous selection of lipids was made according to the following features: natural origin, theoretical capability to solubilize lidocaine, melting point and availability of highly purified samples. The chosen melting point range for solid lipids was 45 to 70 °C (selecting cetyl palmitate and beeswax), to guarantee a solid state in room or body changes in temperature and preserve drug integrity upon heat degradation^25^. The chosen melting point range for liquid lipids was below 0 °C to guarantee a liquid state in room/refrigerator changes in temperature (selecting castor, sesame, cottonseed, corn, and capric/caprylic oils). The maintenance of the original physical states assures that these physical transitions do not interfere in the physicochemical outcomes among different formulations, masking possible interaction effects. Liponate GC^®^ is not a super refined oil, but we kept it as a gold standard, since it is the most used liquid lipid to produce NLCs^27^.

### Lidocaine solubility

LD solubility was evaluated as previously reported (adapted method described by Joshi and Patravale^28^). Briefly, increments of 10 mg of LD were added into 500 μL of oil until there was no complete solubility of the added amount. To assess solubility in solid lipids, increments of LD were added to 50 mg of melted solid lipid, and the determination was made visually.

### Partition coefficient of lidocaine

The partition coefficient (log P) of LD in lipids was determined by adding 10 mg of the drug into a mixture of 3 mL of water and 3 mL of lipid and mixed for 24 hours. After that, the aqueous phase was obtained through centrifugation (5000 rpm, 10 minutes) and filtered (0.45 µm pore membrane). LD determination in aqueous phase was obtained in HPLC (refer to LD quantification method)^29^. The determination of LD in the oily phase was obtained through the difference between LD added and LD in the aqueous phase. The partition coefficient was calculated according to Eq. (1):

1 $$logP=\frac{[{\rm{LD}}\,{\rm{in}}\,{\rm{oily}}\,{\rm{phase}}]}{[{\rm{LD}}\,{\rm{in}}\,{\rm{aqueous}}\,{\rm{phase}}]}$$

### Preparation of nanostructured lipid carriers (NLCs)

NLCs were prepared using the hot emulsification-ultrasonication method^30^. Briefly, the lipid phase was melted in a water bath 10 °C above the melting point of the solid lipid, followed by the addition of LD using magnetic stirring up to complete homogenisation. In another beaker, the aqueous phase (containing water and surfactant) was heated using magnetic stirring and dropped into the lipid phase using high-speed agitation (1200 rpm for 3 min in an ultra-turrax blender (IKA^®^ T18 basic, Staufen, Germany)). After this, the emulsion was submitted to tip sonication (Vibracell, Sonics & Materials Inc., Danbury, USA), operated at 130 W potency, 20 kHz frequency and 50% amplitude in cycles of 30 seconds (on/off) for 30 minutes. The formulations were cooled in an ice bath until reaching room temperature and were then stored at room temperature.

### Lidocaine quantification and entrapment efficiency

The entrapment efficiency (EE) of LD by NLC was determined indirectly by an ultrafiltration method using centrifugal filter tubes (Millex, Millipore, Bedford, MA, USA) with a 30 kDa molecular weight cut-off. NLC suspensions were centrifuged at 18514 x g, for 20 min. EE was calculated by the difference between the amount of LD in the formulations and the amount detected in the filtrate, applying Eq. (2)^31,32^:

2 $$E{E}_{LD}=\frac{{\rm{Total}}\,{\rm{amount}}\,{\rm{of}}\,{\rm{LD}}-{\rm{Amount}}\,{\rm{of}}\,{\rm{free}}\,{\rm{LD}}}{{\rm{Total}}\,{\rm{amount}}\,{\rm{of}}\,{\rm{LD}}}100$$

The assay of LD was performed by HPLC as described in its United States Pharmacopeia (USP) monograph^18^, using the Waters Breeze 2 HPLC system (Waters, Milford, Massachusetts, USA). The parameters were: UV detection at 254 nm (detector UV 2998 waters); mobile phase with 4 parts glacial acetic acid 0.5% in deionized water, pH 3.4 and 1 part acetonitrile; flow rate 1.5 mL/min; injection volume 20 µL; column NST 18–300 mm × 3.9 mm × 4 µm (L1); 10-minute running time.

### Determination of hydrodynamic diameter (z-average size), dispersity (PDI) and zeta potential (ζ)

Z-average size (ZA) was determined by Dynamic Light Scattering (DLS) (Zetasizer Nano ZS, Malvern Instruments Ltd, Malvern, England), at a 90° angle and 25 °C, with samples diluted to 1:200 in ultrapure water (refraction index 1.333 – viscosity 0.8905 cP) for adequate correlation coefficient (between 0.7–1). The Zeta potential (ZP) of these diluted samples was determined by electrophoretic mobility with the same instrument.

### Differential Scanning Calorimetry (DSC)

DSC thermograms of bulk materials and a sample of NLCs were obtained using a Mettler instrument model DSC1 (Mettler-Toledo Schwerzenbach, Switzerland). The samples were weighted on microanalytical balance Metler Toledo, model MX5 (Mettler-Toledo Schwerzenbach, Switzerland). Blanks were automatically deducted. For analysis the parameters were aluminium pans, with a temperature range of 20–80 °C, and heating rate of 5 °C per minute, N_2_ atmosphere (50 mL per minute).

### Lidocaine *in vitro* release profile

The release of LD was analysed in a Franz diffusion cell system, in which a dialysis membrane with a molecular exclusion pore size of 14 kDa separating the donor (1 mL of sample) and acceptor (12 mL of phosphate buffer pH 7.4) compartments. The cells were kept at 37 °C under magnetic stirring (300 rpm)^33^. 200 µL of each sample at each time point was withdrawn from the acceptor compartment for LD quantification; the same withdrawn volume was replaced by buffer to maintain the total cell volume.

### Transmission electron microscopy

Transmission electron microscopy (TEM) was used to evaluate the morphology, integrity and size of NLCs. Uranyl acetate (2%) was added to appropriately diluted NLCs to provide contrast, followed by a deposit onto copper grids coated with carbon film and dried at room temperature. After drying, micrographs of the samples were obtained, using a JEOL1200 EXII microscope operated at 80 kV^33^.

### Design of Experiment (DoE)

10 factors were used in this experiment: 9 excipients (solid lipids: CP and BW; liquid lipids: CA, CC, CS, CO and SO; surfactants: PS and KO) and one drug model (LD). Hence, the DoE used was the first 10 columns of Hall’s design^34^, which is a balanced DoE: 8 low levels and 8 high levels for each factor distributed in 16 samples without confounding the interactions (Supplementary Fig. S1). We performed an experimental plan made up of two sequential experiments. In experiment 1 (Supplementary Table S1), we fabricated 16 samples based on the excipient proportions published in the research literature. After an initial analysis of the tendencies on the ZA, PDI, ZP, and EE outputs, the range of excipient concentrations was changed, obtaining 16 new samples with similar total mass (experiment 2, Supplementary Table S2). This change of levels from the first to the second experiment aimed to clear up the excipient’s behaviour (increasing or decreasing the outputs’ values) and it relied on the experimenter’s expectations regarding the best proportions among the excipients, given the data from experiment 1. Since LD was the drug model, its levels remained the same for both experiments. Finally, the experiment outputs were submitted to a multilevel analysis.

### Statistical analysis

**Figure 6:**
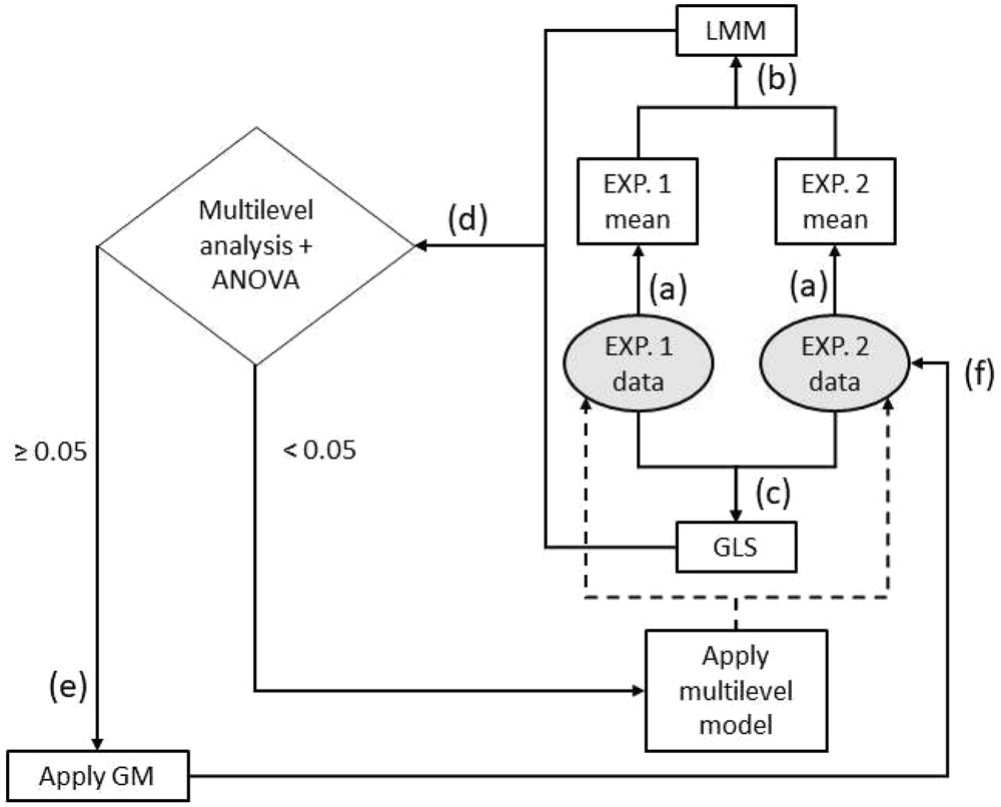
Representation of the first and second steps of the statistical analysis. The sequence of steps is organized by letters (**a**) to (**f**).

The statistical analysis of data obtained in 5.11 was performed in three steps. The first step was the visualization of the mean values of the factors by mean-level graphs, displayed separately according to experiments 1 and 2 (Supplementary Fig. S2), and a statistical descriptive analysis (Table 2). Still in this descriptive analysis, Figs 2(a), 3(a) and 4(a) exhibit the evolution of means of the factors throughout both experiments. We used an arithmetic mean to obtain the mean values of high and low of each factor level (Fig. 6(a)).

The second step was a multilevel analysis. Since the values of high and low levels of the excipients changed from the first to the second experiment, it was necessary to evaluate whether the behaviour of the factors between experiments could be modelled with a simple linear regression or a multilevel model. In Fig. 6(b), LMM is the Linear Multilevel Model generated by only the intercept of both experiments (the means among all the factors). LMM considered that data was generated from two different levels, namely, experiments 1 and 2. In Fig. 6(c), GLS is a Generalized Least Square data model of both experiments together. Then an ANOVA to test the similarity between LMM and GLS was performed (Fig. 6(d)). If they are not statistically different in respect to their mean values (p ≥ 0.05), we can model the data of all 32 samples with a linear regression. This is the General Model (GM) indicated in Fig. 6(e). If LMM and GLS are statistically different models (p < 0.05), this means that too many changes were made in the high and low levels of the second experiment, and so a multilevel model is needed – the experimenter should try to avoid this situation in order to simplify the analysis.

The third step was interaction analysis. Since the multilevel analysis showed that a simple linear model is adequate to describe the data, GMs can be applied to analyse the interactions among the factors in experiment 2 (Fig. 6(f)). In the Supplementary Material, the GMs for each output indicate the main factors and interactions among them. Then, we can define the graphs of mean-interaction (see Figs 2(b–e), 3(b–e) and 4(b–e)). These graphs are defined by the equations Eq. 3 and Eq. 4^16^:

3 $${{\rm{Mean}}}_{{\rm{L}}}({{\rm{Factor}}}_{{\rm{Y}}})=({{\rm{L}}}_{1}({{\rm{Factor}}}_{{\rm{Y}}}|{{\rm{Factor}}}_{{\rm{X}}})+\cdots +{{\rm{L}}}_{4}({{\rm{Factor}}}_{{\rm{Y}}}|{{\rm{Factor}}}_{{\rm{X}}}))/4$$

4 $${{\rm{Mean}}}_{{\rm{H}}}({{\rm{Factor}}}_{{\rm{Y}}})=({{\rm{H}}}_{1}({{\rm{Factor}}}_{{\rm{Y}}}|{{\rm{Factor}}}_{{\rm{X}}})+\cdots +{{\rm{H}}}_{4}({{\rm{Factor}}}_{{\rm{Y}}}|{{\rm{Factor}}}_{{\rm{X}}}))/4$$

where each L_i_ (Factor_Y_|Factor_X_) is the response value for factor *Y* given that when factor *X* is at its low level and each H_i_ (Factor_Y_|Factor_X_) is the response value for factor *Y*, given factor *X* is at its high level.

The statistical analyses were performed with the language R^35^. We tested all the statistical assumptions of the models defined (Cf. Supplementary information p. 10–15). The normality of the residuals was assessed by the Shapiro-Wilk test. For independence of the errors, the Durbin-Watson test was used. Homoscedasticity was assessed by the Breusch-Pagan test. The analysis of sample influence was carried out with Cook’s distance and hat-values, and coefficient collinearity with the variance inflation factor (VIF). Finally, the confidence interval was checked^36,37^.

## Supplementary information

Supplementary information
