# Supplementary material for: Excipient-excipient interactions in the development of nanocarriers: an innovative statistical approach for formulation decisions
Source: Sci Rep. 2019 Jul 24;9:10738. doi: 10.1038/s41598-019-47270-w (PMC6656889; doi:10.1038/s41598-019-47270-w)
Supplement: Supplementary file 1 — Supplementary information [file 41598_2019_47270_MOESM1_ESM.pdf]

Excipient-excipient interactions in the development of nanocarriers: an innovative statistical approach for formulation decisions

Viviane Lucia Beraldo-de-Araújo[1,2,*], Anderson Beraldo-de-Araújo[3], Juliana Souza Ribeiro Costa[1,2], Ana Carolina Martins Pelegrine[2], Lígia Nunes Moraes Ribeiro[1], Eneida de Paula[1], Laura Oliveira-Nascimento[2]

[1]Department of Biochemistry and Tissue Biology, Biology Institute, State University of Campinas, Brazil, Rua Monteiro Lobato, 255, Campinas, SP, Brazil, Postal Code 13083-862;

[2]Pharmaceutical Technology Laboratory, Faculty of Pharmaceutical Sciences, State University of Campinas, Brazil, Rua Candido Portinari, 200, Campinas, SP, Brazil, Postal Code: 13083-871;

[3]Center for Natural and Human Sciences, Federal University of ABC, Santo André, Brazil, Postal Code: 05508-090.

* Corresponding author: vivi.beraldo@gmail.com

Supplementary Information

Table S1: Design of Experiment of experiment 1

Table S1. Hall's design of experiment 1 (Factors: CP=cetyl palmitate, BW=beeswax, CC=capric/caprylic oil, SO=sesame oil, KO=poloxamer 188, CS=cottonseed oil, CO=corn oil, CA=castor oil, PS=polysorbate 80, LD=lidocaine. The values on each column refers to the amount of excipient, in milligrams, in each formulation. Formulations: NLC#1_1 refers to the first formulation of experiment 1, and so on to the subsequent formulations).

**Factors**										
**Formulation**	1	2	3	4	5	6	7	8	9	10
	CP	BW	CC	SO	KO	CS	CO	CA	PS	LD
**NLC#1_1**	290	290	10	10	85	10	10	10	135	25
**NLC#2_1**	450	290	10	10	135	50	50	10	85	50
**NLC#3_1**	290	450	10	10	135	50	10	50	85	25
**NLC#4_1**	450	450	10	10	85	10	50	50	85	25
**NLC#5_1**	290	290	50	10	135	10	50	50	135	50
**NLC#6_1**	450	290	50	10	85	50	10	50	135	50
**NLC#7_1**	290	450	50	10	85	50	50	10	135	25
**NLC#8_1**	450	450	50	10	135	10	10	10	85	50
**NLC#9_1**	290	290	10	50	85	50	50	50	85	50
**NLC#10_1**	450	290	10	50	135	10	10	50	135	25
**NLC#11_1**	290	450	10	50	135	10	50	10	135	50
**NLC#12_1**	450	450	10	50	85	50	10	10	135	50
**NLC#13_1**	290	290	50	50	135	50	10	10	85	25
**NLC#14_1**	450	290	50	50	85	10	50	10	85	25
**NLC#15_1**	290	450	50	50	85	10	10	50	85	50
**NLC#16_1**	450	450	50	50	135	50	50	50	135	25

Table S2: Design of Experiment of experiment 2

Table S2. Hall's design of experiment 2 (Factors: CP=cetyl palmitate, BW=beeswax, CC=capric/caprylic oil, SO=sesame oil, KO=poloxamer 188, CS=cottonseed oil, CO=corn oil, CA=castor oil, PS=polysorbate 80, LD=lidocaine. The values on each column refers to the amount of excipient, in milligrams, in each formulation. Formulations: NLC#1_2 refers to the first formulation of experiment 2, and so on to the subsequent formulations).

**Factors**										
**Formulation**	**1**	**2**	**3**	**4**	**5**	**6**	**7**	**8**	**9**	**10**
	**CP**	**BW**	**CC**	**SO**	**KO**	**CS**	**CO**	**CA**	**PS**	**LD**
**NLC#1_2**	190	190	10	10	100	10	10	10	200	25
**NLC#2_2**	390	190	10	10	200	90	90	10	100	50
**NLC#3_2**	190	390	10	10	200	90	10	90	100	25
**NLC#4_2**	390	390	10	10	100	10	90	90	100	25
**NLC#5_2**	190	190	90	10	200	10	90	90	200	50
**NLC#6_2**	390	190	90	10	100	90	10	90	200	50
**NLC#7_2**	190	390	90	10	100	90	90	10	200	25
**NLC#8_2**	390	390	90	10	200	10	10	10	100	50
**NLC#9_2**	190	190	10	90	100	90	90	90	100	50
**NLC#10_2**	390	190	10	90	200	10	10	90	200	25
**NLC#11_2**	190	390	10	90	200	10	90	10	200	50
**NLC#12_2**	390	390	10	90	100	90	10	10	200	50
**NLC#13_2**	190	190	90	90	200	90	10	10	100	25
**NLC#14_2**	390	190	90	90	100	10	90	10	100	25
**NLC#15_2**	190	390	90	90	100	10	10	90	100	50
**NLC#16_2**	390	390	90	90	200	90	90	90	200	25

Table S3. Mean outputs of Experiment 1.

Table S3. Outputs of experiment 1 (Outputs: z-average ± SD (nm), PDI ± SD, Zeta potential ± SD (mV) and EE. Formulations: NLC#1_2 refers to the first formulation of experiment 2, and so on to the subsequent formulations).

Formulation	Outputs						
	Z-AVERAGE (nm)	± SD (nm)	PDI	± SD	ZETA POTENCIAL (mV)	± SD (mV)	EE
**NLC#1_1**	300	6	0,23	0,02	-42,0	0,3	77,4
**NLC#2_1**	352	5	0,27	0,03	-51,3	0,1	69,7
**NLC#3_1**	304	7	0,24	0,03	-47	1	75,3
**NLC#4_1**	401	7	0,35	0,06	-49,7	0,6	72,8
**NLC#5_1**	273	6	0,24	0,01	-48,7	0,8	71,5
**NLC#6_1**	336	8	0,22	0,02	-48,5	0,7	74,1
**NLC#7_1**	344	5	0,16	0,09	-42,9	0,9	75,0
**NLC#8_1**	397	5	0,41	0,03	-50	1	74,7
**NLC#9_1**	349	3	0,28	0,01	-49,4	0,5	74,2
**NLC#10_1**	327	8	0,25	0,01	-40,7	0,3	38,1
**NLC#11_1**	256	1	0,23	0,01	-48	1	65,9
**NLC#12_1**	334	1	0,24	0,02	-43,2	0,8	71,8
**NLC#13_1**	315	1	0,27	0,01	-48,4	0,3	96,6
**NLC#14_1**	368	6	0,18	0,05	-45,2	0,3	61,6
**NLC#15_1**	264	3	0,25	0,01	-42,7	0,3	61,1
**NLC#16_1**	355	5	0,32	0,03	-37,4	0,7	57,7

Table S4. Mean outputs of Experiment 2.

Table S4. Outputs of experiment 2 (Outputs: z-average ± SD (nm), PDI ± SD, Zeta potential ± SD (mV) and EE. Formulations: NLC#1_2 refers to the first formulation of experiment 2, and so on to the subsequent formulations).

	Outputs						
**Formulation**	**Z-AVERAGE (nm)**	**± SD (nm)**	**PDI**	**± SD**	**ZETA POTENCIAL (mV)**	**± SD (mV)**	**EE**
**NLC#1_2**	146	4	0,33	0,03	-34,9	0,3	68,2
**NLC#2_2**	309	6	0,19	0,05	-49	1	65,3
**NLC#3_2**	341	13	0,18	0,06	-54	2	71,9
**NLC#4_2**	394	28	0,23	0,16	-43,3	0,7	68,3
**NLC#5_2**	268	6	0,21	0,03	-48	2	77,2
**NLC#6_2**	324	6	0,17	0,04	-48	1	78,9
**NLC#7_2**	219	1	0,24	0,01	-41	0,6	74,1
**NLC#8_2**	311	1	0,09	0,07	-48,8	0,2	74,5
**NLC#9_2**	370	7	0,14	0,09	-49	0,9	73,1
**NLC#10_2**	301	2	0,19	0,03	-50	1	75,2
**NLC#11_2**	274	2	0,25	0,01	-45,8	0,3	73,0
**NLC#12_2**	361	8	0,21	0,07	-52,1	0,3	76,9
**NLC#13_2**	255	4	0,23	0,005	-40,2	0,5	71,4
**NLC#14_2**	342	4	0,16	0,04	-45,1	0,8	74,0
**NLC#15_2**	385	15	0,16	0,1	-48,4	0,9	79,4
**NLC#16_2**	338	8	0,2	0,08	-48	1	78,3

Figure S1. Hall's DoE correlation matrix.

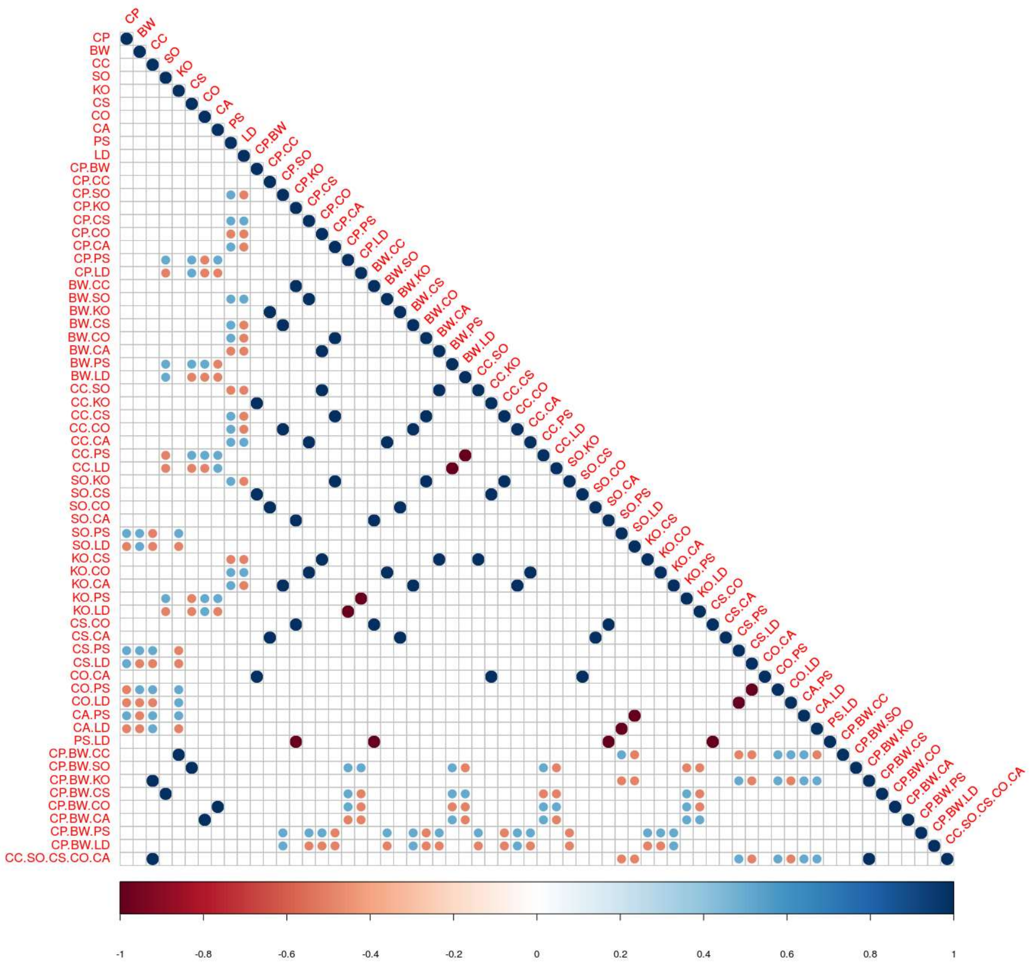

Figure S1. Hall's DoE theoretical correlation matrix. Dark blue dots indicate total positive correlation. Dark red dots indicate total negative correlation. Light color dots indicate intermediary correlation levels.

Figure S2. Graphs of mean-levels.

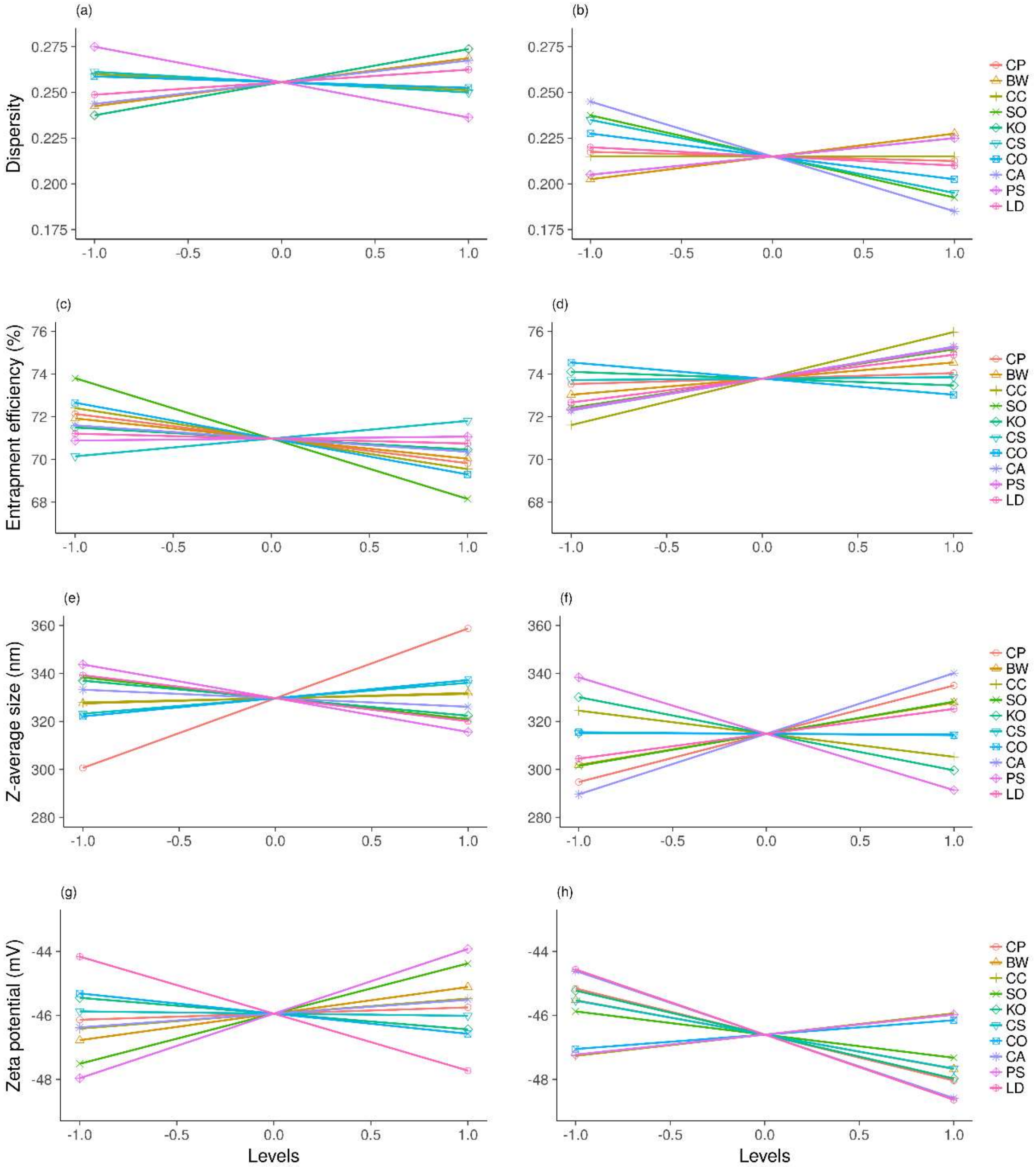

Figure S2. Graphs of mean-level dispersity, entrapment efficiency, z-average size and zeta potential. Variation between low (-1) and high (+1) levels in experiment 1 (a, c, e, g) and experiment 2 (b, d, f, h).

Figures S3. TEM micrographs.

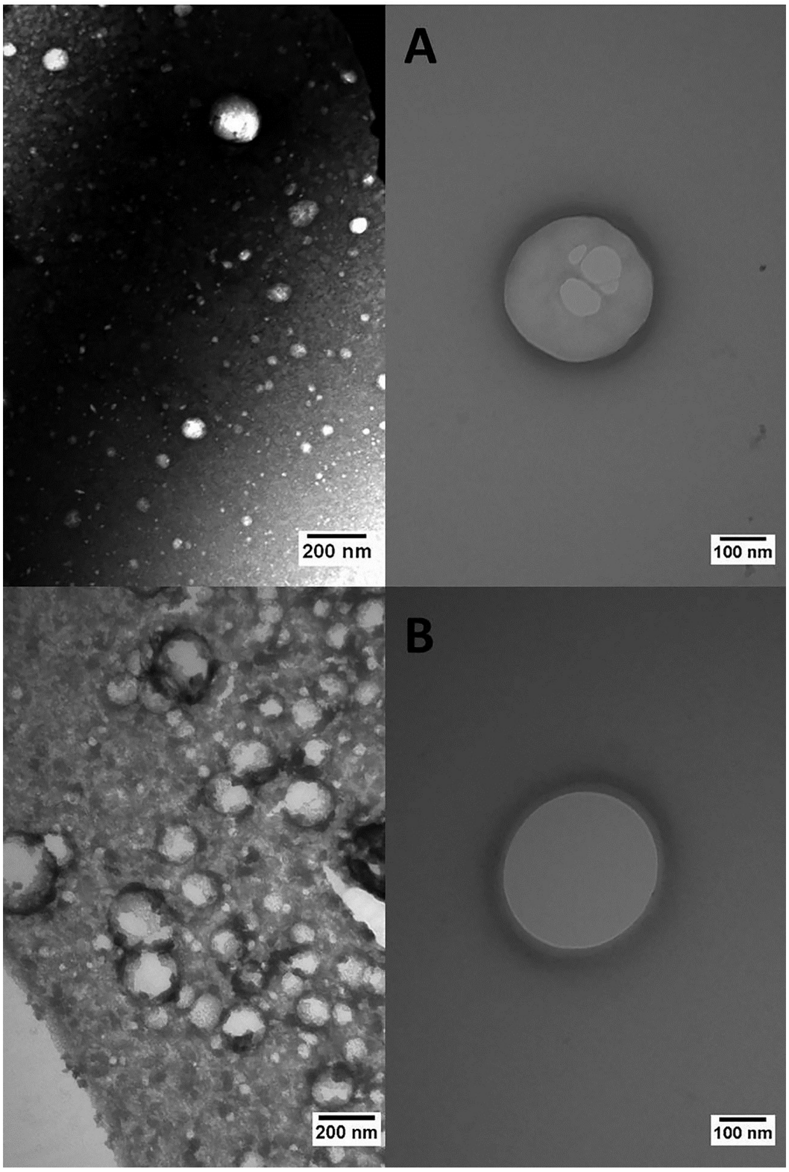

Figure S3. TEM micrographs of empty NLC (A) and LD-loaded NLC (B), both from exp 2. Magnification: 60,000 (left) and 100,000 (right side).

Statistical Models (General Model (GM), General Model applied to experiment 2 (GM applied to exp 2), General Least Square (GLS) and Linear Multilevel Model (LMM)) and ANOVA for each physicochemical output (z-average, dispersity and entrapment efficiency):

--------------Z-AVERAGE-------------

GM

```
Call:
lm.default(formula = scale(average) ~ CA + CP + PS + SO + CA:CP +
    CP:SO + CA:LD + CP:SO + PS:KO, data = design)



Residuals:
     Min        1Q      Median       3Q        Max
-0.81024  -0.37251    0.02792   0.29910  0.98690

Coefficients:
              Estimate    Std. Error   t value    Pr(>|t|)
(Intercept) -6.496e+00  1.234e+00   -5.267     2.41e-05 ***
CA           9.368e-02   1.847e-02    5.071     3.91e-05 ***
CP           1.701e-02   2.784e-03    6.110     3.12e-06 ***
PS           1.114e-02   4.601e-03    2.422     0.023737 *
SO           4.950e-02   1.232e-02    4.01      0.000539 ***
CA:CP       -1.710e-04   3.919e-05   -4.361     0.000229 ***
CP:SO       -1.425e-04   3.919e-05   -3.635     0.001385 **
CA:LD       -7.830e-04   2.199e-04   -3.561     0.001662 **
PS:KO       -6.391e-05   1.732e-05   -3.689     0.001213 **
---
Signif. codes:  0 '***' 0.001 '**' 0.01 '*' 0.05 '.' 0.1 ' ' 1

Residual standard error: 0.5184 on 23 degrees of freedom
Multiple R-squared:  0.8006,  Adjusted R-squared:  0.7312
F-statistic: 11.54 on 8 and 23 DF,  p-value: 1.966e-06
```

GM applied to exp 2

```
Call:
lm.default(formula = scale(average) ~ CA + CP + PS + SO + CA:CP +
    CP:SO + CA:LD + CP:SO + PS:KO, data = design2)

Residuals:
     Min         1Q        Median       3Q         Max
-0.48027    -0.11974    -0.02948    0.20197    0.29161

Coefficients:
               Estimate      Std. Error     t value     Pr(>|t|)
(Intercept)  -7.648e+00    1.648e+00     -4.641      0.00237 **
CA            1.028e-01     2.107e-02      4.882      0.00179 **
CP            1.975e-02     3.808e-03      5.187      0.00127 **
PS            1.477e-02     4.951e-03      2.982      0.02045 *
SO            5.473e-02     1.330e-02     4.114      0.00449 **
CA:CP        -1.989e-04     4.464e-05    -4.456      0.00295 **
CP:SO        -1.596e-04     4.464e-05    -3.575      0.00904 **
CA:LD       -8.256e-04      2.338e-04    -3.531      0.00959 **
PS:KO        -6.352e-05      1.314e-05  -4.834      0.00189 **
---
Signif. codes:  0 '***' 0.001 '**' 0.01 '*' 0.05 '.' 0.1 ' ' 1
```

Residual standard error: 0.335 on 7 degrees of freedom
Multiple R-squared: 0.9476, Adjusted R-squared: 0.8878
F-statistic: 15.83 on 8 and 7 DF, p-value: 0.0007783

GLS

Generalized least squares fit by maximum likelihood
Model: scale(average) ~ 1
Data: design

AIC	BIC	logLik
93.79611	96.72758	-44.89805

Coefficients:

	Value	Std.Error	t-value	p-value
(Intercept)	0	0.1767767	0	1

Standardized residuals:

Min	Q1	Med	Q3	Max
-2.2185950	-0.6182530	0.1765478	0.6545023	1.6909655

Residual standard error: 0.984251
Degrees of freedom: 32 total; 31 residual

LMM

Linear mixed-effects model fit by maximum likelihood
Data: design

AIC	BIC	logLik
95.79611	100.1933	-44.89805

Random effects:
Formula: ~1 | experiment

	(Intercept)	Residual
StdDev:	2.343962e-05	0.984251

Fixed effects: scale(average) ~ 1

	Value	Std.Error	DF	t-value	p-value
(Intercept)	0	0.1767767	30	0	1

Standardized Within-Group Residuals:

Min	Q1	Med	Q3	Max
-2.2185950	-0.6182530	0.1765478	0.6545023	1.6909655

Number of Observations: 32
Number of Groups: 2

ANOVA

Model		df	AIC	BIC	logLik	Test	L.Ratio	p-value
GLS	1	2	93.79611	96.72758	-44.89805			
LMM	2	3	95.79611	100.19332	-44.89805	1 vs 2	1.079874e-08	0.9999

---------------DISPERSITY---------------

GM

lm.default(formula = scale(dispersity) ~ PS + BW + CA + LD +
CP:BW + CP:PS + CA:CP + BW:KO + LD:CO, data = design)

Coefficients:

	Estimate	Std. Error	t value	Pr(>|t|)
(Intercept)	-3.741e-01	5.108e-01	-0.732	0.471751
PS	3.620e-02	5.280e-03	6.856	6.91e-07 ***
BW	-1.475e-02	2.473e-03	-5.965	5.29e-06 ***
CA	-4.041e-02	9.482e-03	-4.262	0.000318 ***
LD	2.959e-02	8.379e-03	3.532	0.001877 **
BW:CP	4.276e-05	6.251e-06	6.840	7.17e-07 ***
PS:CP	-1.368e-04	1.729e-05	-7.913	7.08e-08 ***
CA:CP	3.302e-04	3.142e-05	4.144	0.000424 ***
BW:KO	2.202e-05	6.292e-06	3.500	0.002025 **
LD:CO	-3.433e-04	7.589e-05	-4.523	0.000168 ***

Signif. codes: 0 '***' 0.001 '**' 0.01 '*' 0.05 '.' 0.1 ' ' 1

Residual standard error: 0.4809 on 22 degrees of freedom
Multiple R-squared: 0.8359, Adjusted R-squared: 0.7687
F-statistic: 12.45 on 9 and 22 DF, p-value: 9.51e-07

GM applied to exp 2

lm.default(formula = scale(dispersity) ~ PS + BW + CA + LD +
CP:BW + CP:PS + CA:CP + BW:KO + LD:CO, data = design2)

Coefficients:

	Estimate	Std. Error	t value	Pr(>|t|)
(Intercept)	-3.708e-01	5.236e-01	-0.708	0.505438
PS	3.567e-02	5.160e-03	6.912	0.000454 ***
BW	-1.441e-02	2.830e-03	-5.093	0.002236 **
CA	-2.770e-02	9.307e-03	-2.976	0.024762 *
LD	2.246e-02	1.023e-02	2.196	0.070517 .
BW:CP	4.456e-05	8.169e-06	5.455	0.001580 **
PS:CP	-1.217e-04	1.776e-05	-6.852	0.000476 ***
CA:CP	7.704e-04	3.232e-05	2.384	0.014501 *
BW:KO	1.866e-05	6.398e-06	2.917	0.026737 *
LD:CO	-3.410e-04	7.058e-05	-4.831	0.002908 **

Signif. codes: 0 '***' 0.001 '**' 0.01 '*' 0.05 '.' 0.1 ' ' 1

Residual standard error: 0.3868 on 6 degrees of freedom
Multiple R-squared: 0.9401, Adjusted R-squared: 0.8504
F-statistic: 10.47 on 9 and 6 DF, p-value: 0.0049

GLS

Generalized least squares fit by maximum likelihood
Model: scale(dispersity) ~ 1
Data: design

AIC	BIC	logLik
93.79611	96.72758	-44.89805

Coefficients:

	Value	Std.Error	t-value	p-value
(Intercept)	-8.341116e-17	0.1767767	-4.718448e-16	1

Standardized residuals:

Min	Q1	Med	Q3	Max
-1.6483116	-0.7836235	-0.0918731	0.3404709	2.3292534

Residual standard error: 0.984251
Degrees of freedom: 32 total; 31 residual

LMM

Linear mixed-effects model fit by maximum likelihood
Data: design

AIC	BIC	logLik
95.14171	99.53891	-44.57085

Random effects:
Formula: ~1 | experiment

	(Intercept)	Residual
StdDev:	0.250853	0.9517472

Fixed effects: scale(dispersity) ~ 1

	Value	Std.Error	DF	t-value	p-value
(Intercept)	-1.996321e-16	0.2483921	30	-8.036973e-16	1

Standardized Within-Group Residuals:

Min	Q1	Med	Q3	Max
-1.5381476	-0.6191546	-0.1073979	0.4291332	2.2423448

Number of Observations: 32
Number of Groups: 2

ANOVA

Model		df	AIC	BIC	logLik	Test	L.Ratio	p-value
GLS	1	2	93.79611	96.72758	-44.89805			
LMM	2	3	95.14171	99.53891	-44.57085	1 vs 2	0.6544013	0.4185

--------------ENTRAPMENT EFFICIENCY---------------

GM

lm.default(formula = scale(entrapment) ~ CA + SO + PS + CC +
CO + CP + CO:CP + CO:BW + CS:CC + CS:PS + SO:CC + CA:CP +
CA:BW + CO:PS, data = design)

Coefficients:

	Estimate	Std. Error	t value	Pr(>|t|)
(Intercept)	9.269e+00	2.204e+00	4.206	0.000594 ***
CA	6.142e-02	2.280e-02	2.694	0.015370 *
SO	-8.651e-02	1.214e-02	-7.125	1.70e-06 ***
PS	-4.604e-02	9.411e-03	-4.892	0.000137 ***
CC	-1.851e-02	8.120e-03	-2.280	0.035781 *
CO	-9.966e-02	4.025e-02	-2.476	0.024108 *
CP	-7.309e-03	3.777e-03	-1.935	0.069765 .
CO:CP	-2.410e-04	6.954e-05	-3.466	0.002951 **
CO:BW	3.184e-04	5.569e-05	5.717	2.52e-05 ***
CC:CS	-1.891e-03	2.803e-04	-6.746	3.43e-06 ***
PS:CS	6.403e-04	9.200e-05	6.959	2.30e-06 ***
SO:CC	1.907e-03	2.585e-04	7.376	1.08e-06 ***
CA:CP	3.520e-04	7.465e-05	4.715	0.000200 ***
CA:BW	-5.313e-04	8.094e-05	-6.563	4.83e-06 ***
PS:CO	4.962e-04	1.304e-04	3.805	0.001415 **

Signif. codes: 0 '***' 0.001 '**' 0.01 '*' 0.05 '.' 0.1 ' ' 1

Residual standard error: 0.5242 on 17 degrees of freedom
Multiple R-squared: 0.8493, Adjusted R-squared: 0.7252
F-statistic: 6.843 on 14 and 17 DF, p-value: 0.0001695

GM applied to exp 2

lm.default(formula = scale(entrapment) ~ CA + SO + PS + CC +
CP + CS:CC + CS:PS + CP:SO + CP:CS + CP:PS, data = design2)

Coefficients:

	Estimate	Std. Error	t value	Pr(>|t|)
(Intercept)	-5.292e-01	1.168e+00	-0.453	0.669425
CA	4.682e-03	2.019e-03	2.319	0.068127 .
SO	1.998e-02	5.599e-03	3.568	0.016080 *
PS	-1.375e-02	6.381e-03	-2.155	0.083760 .
CC	2.569e-02	2.319e-03	11.078	0.000104 ***
CP	-7.867e-03	3.429e-03	-2.295	0.070250 .
CC:CS	-3.055e-04	4.219e-05	-7.243	0.000783 ***
PS:CS	1.408e-04	3.193e-05	4.410	0.006957 **
SO:CP	-5.550e-05	1.835e-05	-3.024	0.029286 *
CP:CS	-3.550e-05	1.397e-05	-2.542	0.051795 .
PS:CP	7.776e-05	2.272e-05	3.422	0.018789 *

Signif. codes: 0 '***' 0.001 '**' 0.01 '*' 0.05 '.' 0.1 ' ' 1

Residual standard error: 0.2296 on 5 degrees of freedom
Multiple R-squared: 0.9824, Adjusted R-squared: 0.9473
F-statistic: 27.94 on 10 and 5 DF, p-value: 0.000914

GLS

Generalized least squares fit by maximum likelihood
Model: scale(entrapment) ~ 1
Data: design

AIC	BIC	logLik
93.79611	96.72758	-44.89805

Coefficients:

	Value	Std.Error	t-value	p-value
(Intercept)	- 3.925231e-	16 0.1767767	-2.220446e-15	1

Standardized residuals:

Min	Q1	Med	Q3	Max
-2.5828828	-0.3453052	0.2237578	0.5884000	1.5497297

Residual standard error: 0.984251
Degrees of freedom: 32 total; 31 residual

LMM

Linear mixed-effects model fit by maximum likelihood
Data: design

AIC	BIC	logLik
95.55563	99.95284	-44.77782

Random effects:
Formula: ~1 | experiment

	(Intercept)	Residual
StdDev:	0.1876618	0.9661951

Fixed effects: scale(entrapment) ~ 1

	Value	Std.Error	DF	t-value	p-value
(Intercept)	-2.982381e-16	0.2197509	30	-1.357164e-15	1

Standardized Within-Group Residuals:

Min	Q1	Med	Q3	Max
-2.5119893	-0.3775700	0.2279392	0.6566477	1.4595290

Number of Observations: 32
Number of Groups: 2

ANOVA

	Model	df	AIC	BIC	logLik	Test	L.Ratio	p-value
GLS	1	2	93.79611	96.72758	-44.89805			
LMM	2	3	95.55563	99.95284	-44.77782	1 vs 2	0.2404768	0.6239
